# Unexpected Expression and Function of FcεRI in Immortalized Breast Cancer Cells: A Cautionary Null Study

**DOI:** 10.3390/cells13161399

**Published:** 2024-08-22

**Authors:** Alexandria M. Ashbaugh, David O. Lyons, Carianna M. Keyser, Nicholas A. Pullen

**Affiliations:** 1 Department of Chemistry and Biochemistry, University of Northern Colorado, Greeley, CO 80639, USA; ashb7394@bears.unco.edu; 2 Department of Biological Sciences, University of Northern Colorado, Greeley, CO 80639, USA; lyon1016@bears.unco.edu (D.O.L.); carianna.keyser@unco.edu (C.M.K.)

**Keywords:** mast cell, breast cancer, FcεRI

## Abstract

The high-affinity IgE receptor, FcεRI, is typically associated with type 2 effectors such as mast cells (MC). The relatively unique expression profile of FcεRI and accumulating evidence from pre-clinical and clinical settings, such as MC interactions with tumors, have led us to study MCs as a potential therapeutic target in breast cancer. Our work identified MCs interacting with tumor cells at primary sites using the 4T1 (BALB/c) adenocarcinoma model in vivo. However, this analysis was complicated by a surprising finding that the tumor cells intrinsically and strongly expressed FcεRI. We further studied the expression and function of FcεRI in breast cancer cells in vitro. The 4T1 cells expressed FcεRI to a level similar to mouse bone marrow-derived MC (BMMC). Additionally, two established breast cancer cultures derived from human T-47D cells, one estrogen-dependent (E3) and the other estrogen-withdrawn (EWD8), also expressed FcεRI with EWD8 cells showing the greatest abundance. Functional analyses indicated that IgE-mediated antigen stimulation did not elicit classic Ca^2+^ flux in breast cancer cells as seen in the respective species’ MCs; however, FcεRI crosslinking could stimulate IL-6 production from the T-47D derivatives. Preliminary analysis of primary breast cancer biopsy datasets using R2: Genomics Analysis and Visualization Platform was discordant with our in vivo model and in vitro observations. Indeed, FcεRI mRNA abundance declined in metastatic breast cancers compared to non-cancerous breast tissue. Altogether, we report a previously unidentified and immunologically substantive difference between breast cancer models and human primary tumors. Investigators pursuing FcεRI-relevant therapeutics in this context should be aware of this translational barrier.

## 1. Introduction

Breast cancer (BC) is one of the deadliest diagnosed cancers in the United States, killing upwards of 50,000 women each year [1]. Substantive advances have been made in the prevention and treatment of BC; however, there is room for further development of targeted therapeutics. Mast cells (MC) have been found in and around BC tumors, but their exact function there is unknown [2]. This study sought to observe the effects of MCs, and more specifically of FcεRI, and their roles within the tumors. FcεRI is a specific receptor to MCs in solid tissues and can, therefore, be highly targetable for novel cancer therapeutics where MCs are involved. Basophils also constitutively express FcεRI, but these cells are typically restricted to the blood absent strong Type 2 (or T_h_2) immune activation (e.g., helminth and immediate hypersensitivity reactions). Eosinophils can be induced to express FcεRI by IL-5, typically from subacute to chronic MC and basophil activation and related events. Therefore, as a contextually understudied and highly leverageable target in cancer, we originally sought to characterize the abundance and roles of MCs in an immunocompetent mouse model of metastatic BC, the 4T1 BALB/c model.

Our previous work with the 4T1 model reported that these tumors can skew the host adaptive immune milieu toward a Type 3 (T_h_17) immune module, dependent on tumor cell expression of IL-6 [3]. Other previous work from our lab also identified TGF-β1 as a known stimulus of IL-6 production from MCs [4]. Research in other pathologies, namely fibrotic remodeling of lung and liver, have underscored the role of TGF-β1 as a potent chemokine recruiting MCs, as well as having the ability to induce IL-6 production from MCs. Furthermore, it is well-established that high-grade cancers often accumulate mutations proffering a selective advantage for cancer cell survival in an immunological context, also known as cancer immunoediting [5,6,7,8]. Our prior observations in this model showing an ineffectual T_h_17 polarization, rather than an anti-cancer T_h_1—a concept we term “immuno-distraction”—are in line with the broader processes of immunoediting [3]. In this setting, a particular advantage of cancer cells is their aberrant and concomitant expression of TGF-β1 and IL-6; together these factors are necessary for and reinforce a host T_h_17 phenotype [9,10]. TGF-β1 produced in abundance from tumors can also serve in immune-evasion by inducing local differentiation of T_reg_.

We hypothesized that MCs traffic to high-grade tumors in an IL-6-dependent manner, and that perhaps newly recruited MCs serve as a local, replenishing source of IL-6 and eventual downstream immune cell activation and tumor site-specific fibrosis. While our former position seems to be supported by MC quantitation using a simple staining method in the 4T1 model, the remaining thrust of our original goals were quickly derailed when we started studying FcεRI specifically, which we pre-supposed would only be expressed by MCs. Our investigation revealed that 4T1 cells abundantly expressed FcεRI both in vivo and in vitro, thus rendering this model irrelevant for any investigation seeking to use this receptor as a therapeutic lever. We then hypothesized that this was a characteristic unique to these cells given the relatively restricted expression of FcεRI in a healthy individual. This was found to be false in the context of other commonly used in vitro BC models, where we detected FcεRI protein expression to varying degrees, but mostly similar to that found in MC cultures. RNA expression for all receptor subunits (α, β, γ) was also observed. However, the function of the receptor appears limited compared to normal function in MCs. Finally, we queried several primary human tissue datasets for FcεRI RNA expression, and we saw no substantive FcεRI differences between healthy breast tissue and BC. Ultimately, therefore, we believe that this is a further example of the tenuous translational relevance of such models to real, human breast cancer.

We consider the collected observations presented here to be null data and a cautionary note for the community interested in targeting MCs as a novel cancer immunotherapy. It is important to note that this does not preclude MCs as a ripe target using either directed immune targets, such as FcεRI, or broader modulation with MC stabilizers, such as cromoglicic acid. Rather, we implore our colleagues to exercise heightened caution with certain reductionist research tools. The need to focus on better understanding MCs in the context of cancer is still apparent given the variety of clinical observational reports on their correlative relationships with patient tumors.

## 2. Materials and Methods

### 2.1. Cell Lines

This study used three mouse cell lines and four human cell lines. Mouse cell lines included primary MCs generated in our laboratory using methods previously described [see 4] and that we refer to as NGS3, a 4T1 triple negative BC model, and Jurkat cells (T-cell leukemia), the latter two obtained from ATCC (Manassas, VA, USA). The human cell lines included LAD2 (MC) described in [11] and obtained from the same (Mast Cell Biology Section, Laboratory of Allergic Diseases, NIAID, Bethesda, MD, USA), E3 (ER+ BC), EWD8 (basal-like BC), and MCF7 (ER+ BC). E3 and EWD8 are derivatives of T-47D cells that are described in [12] and obtained from the same; MCF7 cells were obtained from ATCC. We provide NGS3 cells freely to investigators interested in these cells.

### 2.2. Antibodies

The following antibodies were used during the assays performed. The following antibodies were purchased from BioLegend San Diego, CA, USA. Alexa Fluor 488 anti-mouse FcεRIα (Cat No. 134330), APC/Cyanine7 anti-human FcεRIα (Cat No. 334631), purified anti-mouse CD16/32 (Cat No. 101302), and Human TruStain FcX (Cat No. 422302). For immunoblotting monoclonal rabbit anti-FcεRI-alpha, cat. #: A22729 from ABclonal, Woburn, MA, USA, and secondary HRP-conjugated goat anti-rabbit IgG cat. #: 926-80011 from LI-COR Biotechnology, Lincoln, NE, USA.

### 2.3. Chloroacetate Esterase Staining (CAE)

CAE staining was performed for a general quantification of MCs in solid tumors. The CAE kit used was the Naphthol AS-D Chloroacetate Esterase Kit from Sigma-Aldrich, Burlington, MA, USA. Tissue sections were cryosectioned between 8–10 μm and stained according to the manufacturer’s protocol. In a 15 mL tube, 1 mL of sodium nitrite and 1 mL of the fast red violet LB base solutions were mixed via inversion and allowed to stand for 2 min. The solution was then added to 40 mL of DI water warmed to 37 °C, 5 mL of TRIZMAL 6.3 buffer concentrate, and 1 mL of Naphthol AS-D Chloroacetate solution. The solution was thoroughly mixed via inversion and turned to a vibrant red color. Room temperature citrate-acetone-formaldehyde solution was used to fix the samples for 30 s. Slides were rinsed with DI water for 45 s, placed in the chloroacetate solution, and incubated in the dark for 15 min at 37 °C. After incubation, the slides were washed with DI water for 2 min, and then counterstained in hematoxylin for 2 min. The hematoxylin was washed off with tap water, and the slides were allowed to dry before being coverslipped.

Between 8–12 images were taken per section, from individual tumors in 60 female BALB/c mice (n = 30 per group, WT and IL-6KO), ensuring that the center and edges for each section were imaged. MCs were quantified, and data were analyzed based on each sample’s average number of MCs per high-powered field of view [13].

### 2.4. Immunofluorescence

Tumors were collected from BALB/c mice 28 days after injection with 4T1 cells [3]. Tumors were frozen at −80 °C until cryosectioned. Sections were cut 8–10 µm thick and stored at −20 °C until stained and analyzed. The sections were stained with an anti-mouse FcεRIα antibody diluted with 0.2% BSA. Anti-CD16/32 was used for competitive binding to avoid non-specific binding to surface Fcγ receptors on the cancer cells. The samples were incubated at 4 °C for twenty-four hours. After incubation, they were washed with 1X PBS three times. Tumor sections were then counterstained with a 2% Hoechst (nuclei) solution. Hoechst was diluted with 1X PBS. This solution was pipetted onto the samples, and the sections were placed in a dark area for 30 min. After incubation, the Hoechst was washed off with 1X PBS, and the samples were processed for preservation and stored at −20 °C until they were analyzed.

Image capture and analysis were performed using a Zeiss LSM 900 confocal laser scanning microscope and the corresponding software Zen Blue (version 3.4.91). Between 12 and 16 images of each sample were taken at 20× or 40× magnification.

### 2.5. Flow Cytometry 

All prepared samples were evaluated using the Attune NxT acoustic focusing flow cytometer, and all data were analyzed using FlowJo v.10.

#### 2.5.1. FcεRI Flow Cytometry

Samples were prepared using the following protocol adapted from the BioLegend Cell Surface Flow Cytometry Staining Protocol [14]. Cells suspended in complete medium (~1 × 10^6^ cells/mL) were added to 2 mL Eppendorf tubes and centrifuged at 350× *g* for 5 min. Media were discarded. Cells were washed twice with cell staining buffer (BioLegend). Supernatant was discarded. Fc block was added to the pellets and left at 4 °C for 10 min. The antibody (diluted per manufacturer recommendation) and enough cell staining buffer were added to bring the total volume to 100 µL. The samples were incubated again at 4 °C for 20 min. The volume of the tubes was raised using cell staining buffer (~600 µL). Samples were centrifuged at 350× *g* for 5 min. The supernatant was discarded, and samples were washed twice in a similar fashion using cell staining buffer. The supernatant was discarded, and the samples were resuspended in 1 mL of cell staining buffer. Approximately one million cells were analyzed per sample, no-stain controls were compared to isotype to confirm lack of non-specific binding, and cells were otherwise stained using the FcεRIα antibodies.

#### 2.5.2. Ca^2+^ Flux Assay

The Ca^2+^ Flux Assay is a method to assess the degranulation potential of cells using a flow cytometer. This assay was conducted with BMMCs and 4T1s to test the functionality of FcεRI. The cells were sensitized with TNP-KLH (antigen)-specific IgE for twenty-four hours at a 0.5 µg/mL concentration. Fluo-4 AM—the Ca^2+^ fluorescent indicator—was dissolved in DMSO at a concentration of 1 mM. Before the Fluo-4 AM was added to the cells, the cells were centrifuged at 350× *g* for 5 min, and the supernatant was discarded. Cells were washed twice with Ca^2+^-free PBS. Fluo-4 AM was added at 1 µM per 1 × 10^7^ cells/mL. The cells were then incubated for 45 min at 37 °C. After incubation, cells were washed with PBS and allowed to rest for 30 min at room temperature. Flow cytometry was run initially without antigen to obtain a Ca^2+^ baseline for 60 s. Then the sample tube was removed from the flow cytometer and 1 µL of TNP-KLH was added; the lid was closed; sample was mixed via inversion. The tube was immediately placed back on the flow cytometer, and data were collected again for another 60 s to observe a change in calcium concentration [15]. For NGS3 and 4T1 the Ca^2+^ indicator used was Fluo-4 AM from Invitrogen (Waltham, MA, USA). For LAD2, EWD8, E3, and MCF7, the Ca^2+^ indicator was ICR-1 AM from Ion Biosciences (San Marcos, TX, USA). 

### 2.6. Polymerase Chain Reaction (PCR)

PCR was used to determine the cDNA presence of α, β, and γ subunits of FcεRI using primers designed to target the specific subunits. Primers were crosschecked for human and mouse FcεRI (Table 1). All primers were purchased from Invitrogen (Waltham, MA, USA).

#### 2.6.1. RNA Extraction

Total RNA was extracted using the TRIzol Plus RNA Purification Kit from Invitrogen (Waltham, MA, USA). Cell samples were lysed and centrifuged to separate phases, with the liquid phase containing RNA, according to the manufacturer’s protocol. RNA was washed and eluted into a 1.5 mL tube. Purity and concentration were measured with a NanoDrop Spectrophotometer.

RNA without adequate concentrations (lower than 100 ng/mL) and purities (a 260/280 ratio less than 1.5) was further purified; samples were reconstituted in a small volume of liquid. This was accomplished by adding 0.1X volume of 3M sodium acetate (pH 5.2) to the samples, then by adding cold 100% ethanol and cold isopropanol. Tubes were inverted to mix and then placed on ice for 10 min. RNA samples were centrifuged at 14,000× *g* at 4 °C for 10 min. The supernatant was carefully removed, and the pellets were washed with 1000 mL of 75% ethanol. Samples were centrifuged again for 2 min (same settings). The supernatant was removed, and the samples were centrifuged once more for 1 min. Any excess supernatant was removed. The pellets were air-dried for 3 min. The pellets were resuspended with 15mL of nuclease-free water. Purity and concentration were checked once more using the NanoDrop Spectrophotometer (2000/2000c software version 1.6.198).

#### 2.6.2. cDNA Synthesis

This was completed using the QuantiTect Reverse Transcription Kit (Cat. No. 205311) from Qiagen (Germantown, MD, USA). cDNA was made from the RNA samples from all cell lines in a 20 mL volume reaction with 1 mg of total RNA for each cell line. In 0.2 mL nuclease-free PCR tubes, 1mL dNTPs and 1mL anchored oligo dT22 were added and adjusted with water for a total volume of 13 mL. The samples were placed in a thermal cycler at 65 °C for 5 min, then a 12 °C hold. The RNA was placed on ice to allow for denaturation. Then, 4 mL 5X first-strand buffer, 1.2 mL DEPC-treated water, 1 mL DTT, 0.5 mL RNase OUT (Invitrogen 10777-019), and 0.3 mL SuperScript III were added for a total volume of 20 mL. Samples were placed in the thermocycler once more, programmed at 25 °C for 5 min, 50 °C for 1 h, 70 °C for 15 min, and 12 °C hold. Samples were stored at −80 °C until ready for PCR.

#### 2.6.3. PCR

The PCR kit used was the QuantiTect SYBR Green PCR Kit (Cat. No. 204163) from Qiagen (Germantown, MD, USA). Primers were diluted in nuclease-free water for a concentration of 2 mM. Each tube contained 10 μLMasterMix, 1 μL forward primer, 1 μL reverse primer, and 8 μLcDNA per cell line. Samples were loaded into the thermocycler and programmed for 1 cycle of 94 °C for 2 min (initial denaturation), 40 cycles of denaturation at 94 °C for 30 s, annealing at 55 °C for 30 s, and elongation at 72 °C for 30 s, followed by a 10-min extension for final elongation at 72 °C, and finally a 12 °C hold.

#### 2.6.4. Gel Electrophoresis

A 2% agarose gel was prepared. Samples were prepared for the gel by combining 3.3 mL 6X SYBR Green, 4.7 mL nuclease-free water, and 12 mL PCR samples. The samples were loaded alongside a 1 Kb GeneRuler Plus DNA ladder from ThermoFisher Scientific (Waltham, MA, USA). The gel was run in 1X TAE at 100 V until adequate band separation was achieved (about 1 h).

### 2.7. Immunoblotting

Lysates were collected using 1X lysis buffer (Cell Signaling Technology, Danvers, MA, USA), and 50 μg of total protein was loaded per sample into 4–20% polyacrylamide gels (Mini-PROTEAN TGX, Bio-Rad Laboratories, Hercules, CA, USA). Electrophoresis was performed at 100 V for at least 40 min and transferred to 0.2 μm nitrocellulose using the Bio-Rad Trans-Blot Turbo system and the “1 Mini-TGX” pre-programmed protocol. Membrane was then blocked 1 h at ambient with tris-buffered saline (TBS, Bio-Rad 10X stock diluted to 1X) containing 0.01% (*v*/*v*) Tween-20 and 5% (*w*/*v*) bovine serum albumin (BSA, VWR International, Radnor, PA, USA). After washing thrice 5 min each with 1X TBS-Tween (0.01% Tween-20, TBST), membrane was incubated in primary antibody (monoclonal rabbit anti-FcεRI-alpha, cat. #: A22729, ABclonal, Woburn, MA, USA) diluted 1:1000 in blocking buffer overnight at 4 °C. Membrane was then washed thrice and incubated 1 h at ambient in secondary antibody solution: TBST containing 5% (*w*/*v*) non-fat dried milk with HRP-conjugated goat anti-rabbit IgG (cat. #: 926-80011, LI-COR Biotechnology, Lincoln, NE, USA) diluted 1:5000. After a final round of washing, membrane was developed using Pierce ECL Plus (ThermoFisher Scientific, Waltham, MA, USA) and imaged using an Azure 500 System (Azure Biosystems, Dublin, CA, USA).

### 2.8. IL-6 ELISA

This assay was performed with the Human IL-6 ELISA Max Deluxe Set from BioLegend (San Diego, CA, USA), following the manufacturer’s protocol. First, diluted capture antibody at a concentration of 0.5 mg/mL was dispensed at 100 mL in each well of a flat-bottom 96-well plate (high binding). The plate was covered and incubated at room temperature overnight. After the overnight incubation, the plate was washed with wash buffer (0.05% Tween-20 in 1X PBS), and then 300 mL of block buffer (0.5% BSA in 1X PBS) was added to the wells and incubated at room temperature for 1 h. The plate was washed, and 100 mL of the samples and diluted standard solutions were added to the wells and incubated at room temperature for 2 h. The plate was washed, and the biotinylated detection antibody was added at a concentration of 0.5 mg/mL at a volume of 100 mL/well and incubated at room temperature for 1 h. The plate was rewashed, and the avidin-HRP was added to each well (100 mL/well) and incubated for 30 min. Excess avidin-HRP was washed off, and the TMB substrate was added to each well (100 mL/well). The data were collected immediately using a SpectraMax 190 microplate reader. The data were analyzed using GraphPad Prism 10 software.

The mouse IL-6 ELISA Max Deluxe Set from BioLegend San Diego, CA, USA, was completed similarly to the Human IL-6 ELISA steps above.

### 2.9. R-2 Genomic Data Search

The genetic database R2: Genomics Analysis and Visualization Platform http://r2platform.com (accessed on 15 April 2024) was used to apply the data collected with the in vitro models within this study to human tissue removed from patients. The genes GAPDH, ACTB, FceR-1a, and TGF-b1 were searched on the tissue expression data from the following available studies (Table 2).

## 3. Results

### 3.1. CAE Staining

**Figure 1:**
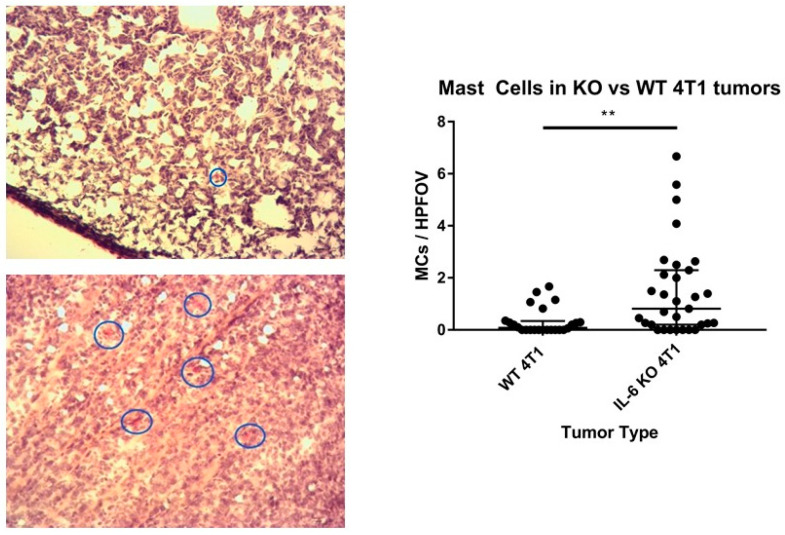
Left: Representative images of CAE staining at 40X objective magnification. Mast cells circled in blue for context. Top: WT 4T1 tumors. Bottom: IL-6 tumors. Right: Quantification of the MC concentration between WT and KO tumors. KO tumors were more likely to have higher MC counts (** *p* < 0.05) [13].

For a previous study focusing on systemic changes in T cells and myeloid cells, we generated 4T1 tumors where IL-6 was specifically deleted only in the 4T1 cells; in other words, IL-6 was intact in the host [3]. Numerous tissues, including the primary tumors from each mouse, were banked for later potential research. For the present work, we turned to those cryopreserved tumor specimens to examine MC presence. Chloroacetate esterase (CAE) staining is a method that stains MC granules magenta/pink while other cells are stained purple (Figure 1). This staining technique was applied to the 4T1 tumor samples, and it was found that IL-6 KO tumors had a higher concentration of intratumoral MCs compared to the WT control tumors [13]. We then sought to characterize the functionality of these MCs more specifically first by examining expression of FcεRI. Thus, immunofluorescent staining with laser scanning confocal microscopy was performed to collect data of cells that expressed only FcεRI, which presumably should be predominantly MCs.

### 3.2. Fluorescence

#### FcεRI in 4T1 Tumors In Vivo

**Figure 2:**
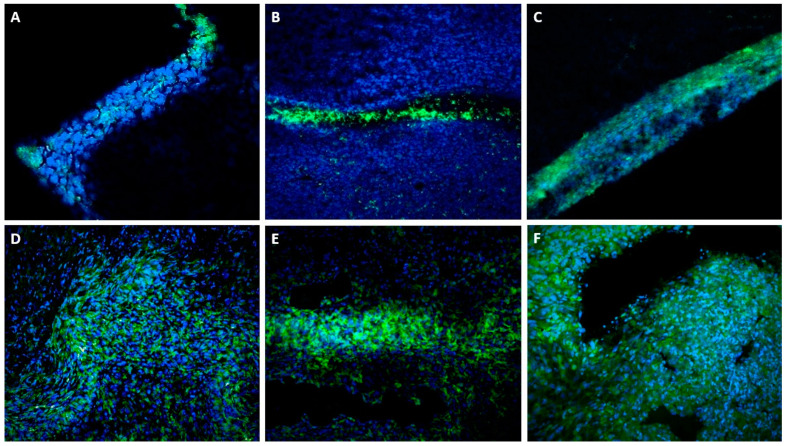
(**A**–**C**) Control “Wildtype” 4T1 tumors stained for FcεRI (green) and Hoechst (blue) at 20X objective magnification. (**D**–**F**) IL-6 knock-out 4T1 tumors stained for FceR-1 (green) and Hoechst (blue) 20X objective magnification. All images were taken with the Zeiss LSM-900 microscope.

Intense FcεRI staining was observed for both the IL-6 KO and WT tumor sections (Figure 2). A few possible reasons exist for a large portion of the positive staining within the tumor samples. Perhaps the tumors contained many MCs, or perhaps other cells expressed the FcεRI. Furthermore, technical errors could also be of concern. Notably, in the WT 4T1 tumors, a fibrous capsule surrounded some tumors where the MC concentration was the highest; this was not seen in the IL-6 KO tumors. Nevertheless, abundant expression of FcεRI was observed regardless of tumor source, with no significant differences. After running through several rounds of troubleshooting and negative controls (different blocking sera, blocking antibodies, antibody titration, isotypes, etc.) we were convinced that the fluorescence detection was real. However, based on the staining pattern, we do not believe this is solely attributable to MCs; rather the 4T1 cells were expressing high levels of FcεRI too.

### 3.3. Anti-FcεRIα Expression In Vitro

**Figure 3:**
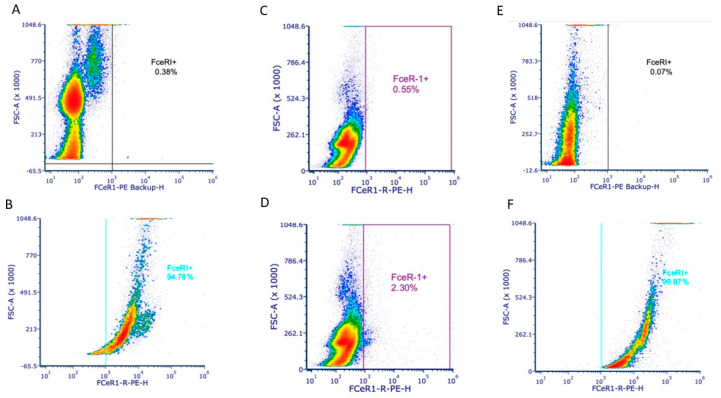
(**A**) Unstained negative control C57BL/6J BMMCs. (**B**) Stained BMMCs for anti-mouse FcεRI, >90% positive, as expected. (**C**) Unstained negative control Jurkat. (**D**) Stained Jurkat for anti-mouse FcεRI. (**E**) Unstained negative control 4T1s. (**F**) Stained 4T1s for anti-mouse FcεRI > 90%, an unexpected result.

To further confirm the expression of FcεRI within solid tumor sections, an in vitro assay was performed using the same FcεRIα antibody used in confocal microscopy. This assay showed a positive result for NGS3 BMMC (a stable MC line generated in our lab) and 4T1 but negative for Jurkat cells, a mouse T-cell leukemia cell line. The positive BMMC and negative Jurkat were expected, though the positive 4T1 were initially unexpected, yet confirmed our observations from in vivo 4T1 tumor studies (Figure 3). A CD16/32 block was used to ensure staining was specific to FcεRI. This expression in the 4T1 cells has not been noted in the literature to our knowledge.

**Figure 4:**
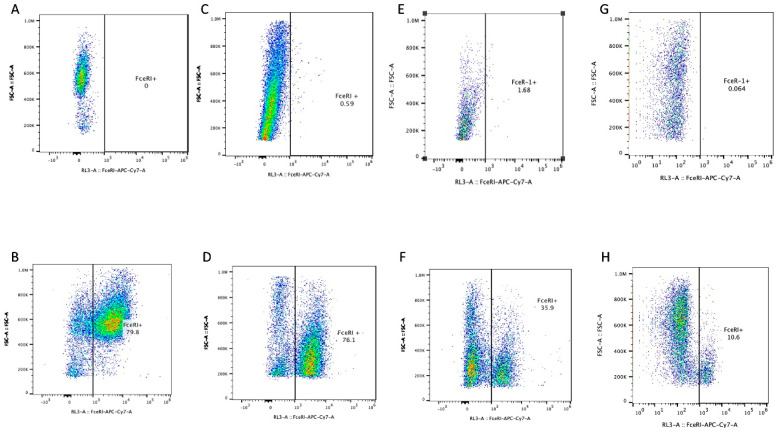
(**A**) Unstained negative control LAD2. (**B**) Stained LAD2 for anti-human FcεRI, ~80% positive as expected. (**C**) Unstained negative control EWD8. (**D**) Stained EWD8 for anti-human FcεRI, ~76% positive, an unexpected result. (**E**) Unstained negative control E3. (**F**) Stained E3 for anti-human FcεRI, ~36% positive, an unexpected result. (**G**) Unstained negative control MCF7. (**H**) Stained MCF7 for anti-human FcεRI, ~11% positive, an unexpected result.

This assay was repeated with human LAD2, E3, EWD8, and MCF7 cells. The results were mostly consistent with the data collected with the mouse cells. The LAD2 cells were used as a positive control and expressed FcεRI as expected. The three breast cancer cell lines also expressed FcεRI. The EWD8s basal-like cell line expressed a similar amount of FcεRI, ~76% positive. This was an unexpected result. The E3 and MCF7 cell lines expressed a lesser amount of FcεRI, ~33% and ~11%, respectively, but still higher than expected for a relatively specific receptor (Figure 4). Both cell lines are ER^+^ luminal-like BC, which tends to be less aggressive than the basal-like EWD8 cell line [12]. This is a possible explanation for the difference in FcεR1 expression. The literature has not noted this expression of FcεRI on human-immortalized BC lines.

### 3.4. Ca^2+^ Flux Assay

**Figure 5:**
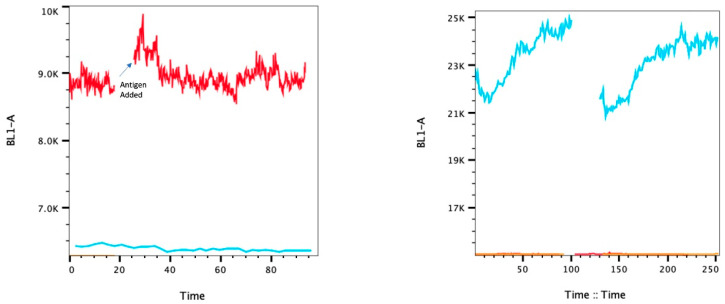
Mouse Cell Ca^2+^ Assay. Left: NGS3 Ca^2+^ (Fluo-4 AM fluorescence). After activation with TNP-KLH, NGS3 increased in Ca^2+^, as expected (red); compared to unstained (blue). Right: 4T1 Ca^2+^ Assay. 4T1 did not have a difference in Ca^2+^ release post-activation with TNP-KLH, however progressively increased in fluorescence as the assay proceeded in analysis when stained (blue); compared to unstained (orange).

Calcium can be used to measure the early activation function of FcεRI. Crosslinking of the receptor via IgE + antigen causes Ca^2+^ release from the endoplasmic reticulum, which is critical for degranulation and can even exit to the ECM [22]. In the mouse model, as expected, the MCs (NGS3) had an increased Ca^2+^ release from the ER into the ECM immediately after adding antigen (Figure 5). The cancer cells did not, with the 4T1s starting low and increasing before and after antigen addition, which could indicate sensitivity to physical manipulation.

**Figure 6:**
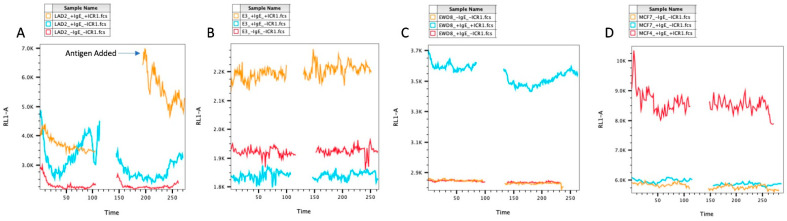
Human Cell Ca^2+^ Assay. (**A**) LAD2 Ca^2+^. (**B**) E3 Ca^2+^. (**C**) EWD8 Ca^2+^. (**D**) MCF7 Ca^2+^. LAD2 showed increased Ca^2+^ after the addition of antigen, as expected. E3, EWD8, and MCF7 showed no change in Ca^2+^ post-antigen stimulation.

In the human model, the LAD2 released Ca^2+^ from the ER after adding antigen, as expected. The E3 and EWD8 cells started at a lower Ca^2+^ level after antigen addition than those without antigen stimulation before increasing slowly. The MCF7s did not show an increase in calcium after adding the antigen (Figure 6).

### 3.5. PCR

**Figure 7:**
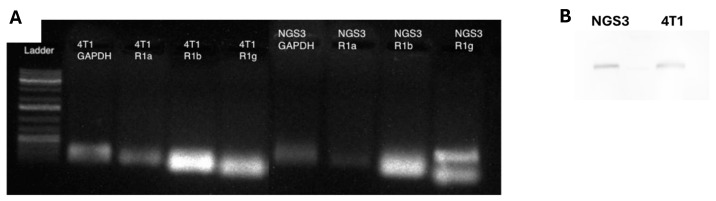
FcεRI subunits expressed in mouse cells lines. (**A**) PCR gel from 4T1 and NGS3 cells (mouse lines). NGS3 expressed all three subunits (αβγ) as expected, and 4T1 expressed all three subunits (αβγ), which was an unexpected result. (**B**) Immunoblot confirming protein expression.

Determining the presence of the α, β, and γ subunits of FcεRI within the 4T1, E3, EWD8, and MCF7 cells is an important step in understanding the function of FcεRI within these cells. The NGS3s expressed all three subunits (αβγ) as expected. Though the γ subunit in the NGS3 showed two bands, the reason for this is unknown and could be a mutation derived from when the cells were immortalized (Figure 7). The 4T1 cells also expressed all three subunits (αβγ), which was initially unexpected but in line with our previous protein results. The β band was brighter in the 4T1 than the β band of the NGS3, which could indicate that the 4T1 produced more of this subunit than the NGS3. However, the point of these assays was simply for binary detection of the receptor subunits (not quantitation), and regardless the 4T1 cells expressed the receptor as confirmed by multiple complementary assays. The expression of the α, β, and γ subunits is a new, translationally confounding, finding that might warrant further study if confirmed in primary human breast cancers.

**Figure 8:**
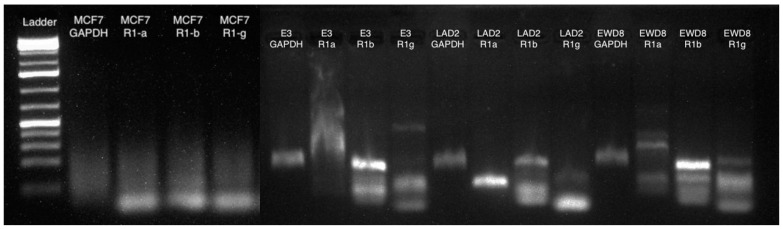
Representative PCR gel from LAD2, E3, EWD8, and MCF7 (human lines). LAD2 expressed all three subunits as expected. E3 and EWD8 expressed all three subunits, an unexpected result. Repeated attempts to detect these subunits at the mRNA level in MCF7 were unsuccessful.

We were able to clearly detect FcεRI (αβγ) subunit mRNA in the E3 and EWD8 cells as was the case for the mouse lines (Figure 8). The brighter β bands in E3 and EWD8 could indicate a potentially higher concentration of this subunit than of the α and β subunits. For the MCF7 cells we have been unable to detect any of the subunits of FcεRI at the mRNA level similar to other cell lines after repeated assays with new cells, and the results seen are most likely primer dimers (Figure 8).

### 3.6. IL-6 ELISA

Activation of FcεRI with IgE and antigen (TNP-KLH) will lead to the upregulation and release of IL-6 from MCs [23]. The ELISA measured the concentration of IL-6 in the medium 24 h after activation with IgE and TNP-KLH to measure the function of the FcεR1. The NGS3s increased the amount of IL-6 after IgE and TNP-KLH activation, though once there was too much stimulation with antigen, IL-6 concentration decreased as expected. The 4T1s did not produce and subsequently release any IL-6 at any concentration of TNP-KLH antigen. This, along with Ca^2+^ data, indicates that the receptor was nonfunctional in the 4T1 cells despite microscopy and flow data observing FcεRI protein and the PCR results indicating all three subunits were transcribed. Looking further down the signaling pathway to determine the difference would allow a better understanding of why/how FcεRI was present on these cells and its overall function.

**Figure 9:**
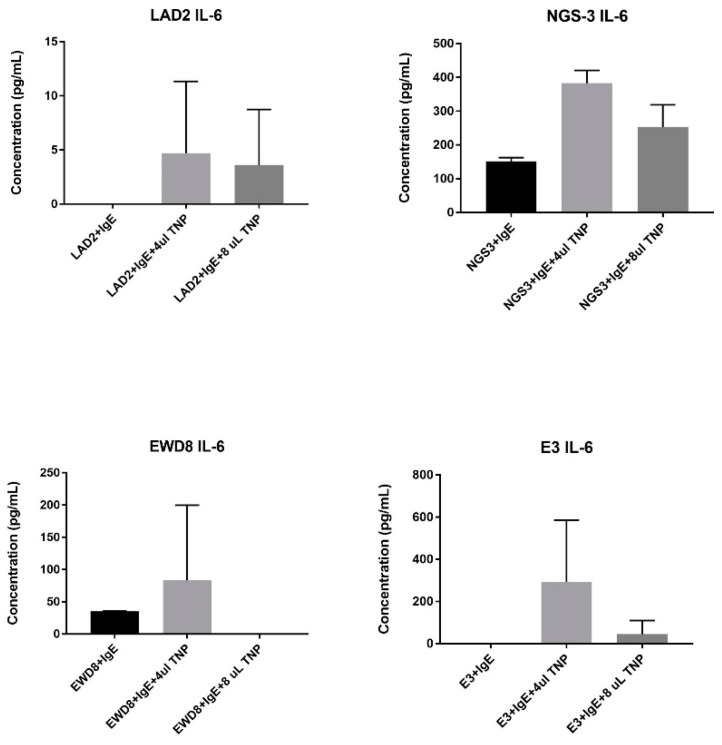
IL-6 ELISA results. LAD2 and NGS3 produced IL-6 as expected when given IgE and antigen. E3 and EWD8 also produced IL-6 which was an unexpected result. 4T1 and MCF7 did not produce any IL-6, and thus are not depicted here.

The human cell lines had a different result. The LAD2s produced and released small amounts of IL-6 when stimulated with IgE and TNP-KLH. The concentration of IL-6 also decreased slightly at the high dose of antigen. This was expected, but typically MCs release more IL-6 at low-dose antigen than was observed here, which could indicate a limitation of our system using a non-human IgE. Though this is a cancerous cell line, it can be assumed that they produced lower amounts of IL-6 for the purposes of this assay. Another interesting observation is that the EWD8s and the E3s produced IL-6, which was unexpected. The EWD8s produced IL-6 with stimulation with IgE and with IgE and antigen. The E3s acted similarly to the LAD2s, with the IL-6 being released after the stimulation with IgE and antigen and decreasing concentrations at the high antigen dose (Figure 9). The MCF7s did not produce any IL-6.

### 3.7. R2 Genomics

**Figure 10:**
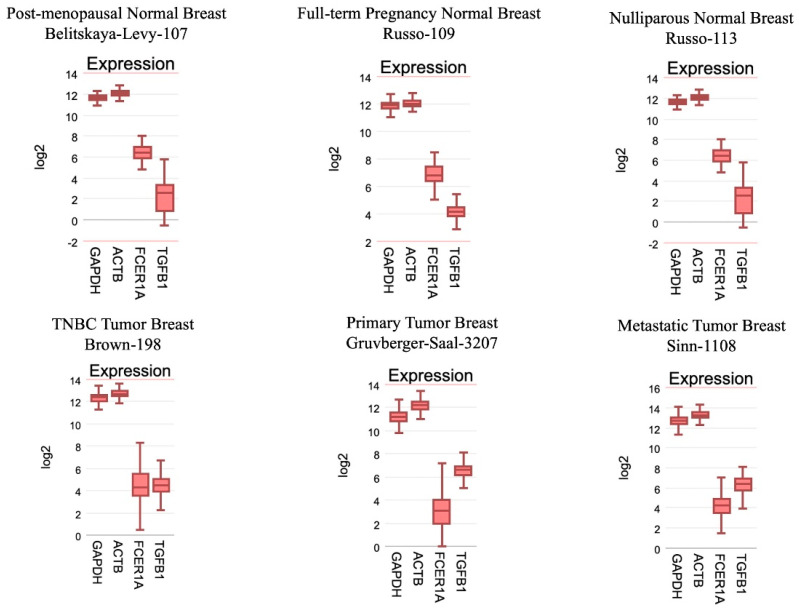
R2 Genomics search results. Top row: normal breast tissue. Bottom row: tumor breast tissue. GAPDH and ACTB from each dataset were included as internal reference standards (i.e., “loading controls”).

Of the six genomic data sets examined, three being normal breast tissue at different life/reproductive stages and three being tumor tissue, there was an overall trend between normal and cancerous: FcεRI was downregulated in a tumor compared to normal tissue. TGF-β is up-regulated in a tumor, consistent with the tumor immunological skewing effect we see in cancer [24]. These differences are seen regardless of studies compared, though it should be noted that these are rough comparisons and should not be treated as matched groups since they are independent, different study sources. Rather these data are illustrative of the following points: FcεR1 being downregulated is an interesting result because it is the opposite of what we see in immortalized cell lines that are commonly used to study BC (Figure 10). This result shows a difference between primary tissue removed from a human patient and the assays conducted with immortalized human cell lines.

## 4. Discussion

TGF-β is a strong chemokine for MCs, with TGF-β being associated with increased MC traffic to fibrotic lesions. These recruited MCs are activated via TGF-β priming and release of IL-6 as seen in lung fibrosis [6,25]. In liver fibrosis MCs stimulated to release tryptase and chymase when near nerves lead to fibrogenesis in chronic liver disease [7] The Francis group from Indiana University aimed to further detail the interplay of MC and TGF-β in contributing to liver fibrosis in a MC-deficient model, and while these results were promising, they have recently been called into question by a retraction [26]. Despite this, we believe there is considerable value in replicating that study as there may be implications for cancer immunology as well.

MCs are most notably known for their role in allergy response (type I hypersensitivity), though their presence in cancer cannot be overlooked [6]. High serum IgE concentration due to allergy has some evidence to support a lower risk of developing chronic lymphocytic leukemia, most likely from the heightened state of the immune system due to the preexisting allergies [27]. In lung adenocarcinoma, mice with high IgE survived longer than mice with low IgE, indicating IgE to be a prognostic factor, the higher the concentration present the better the outcome [28]. Within BC, there is a correlation between low serum IgE (less than 32.6 IU/mL) and the development of cancer [29]. This finding lends evidence to a very probable treatment for BC via IgE immunotherapy [30]. Furthermore, there are a suite of possible FcεRI-targeted therapies that could more specifically target MCs rather than IgE [31].

This study offered null results regarding the utility of the 4T1 model and other in vitro BC lines with respect to MC and FcεRI interrogation. This is important to the scientific community because it leads to less publication bias, presents a more complete view of scientific knowledge, and highlights gaps in research that can be explored [32,33]. The scientific community has made a culture of publishing significant results at the expense of much lost effort in work hours and research dollars toward both irreproducible results and repetitions of null data that were never published (but that probably should have been). [32,33]. Overall, the publication of null results will enhance the efficiency and reliability of scientific research.

From the observations within this research we conclude that: (1) MCs traffic to mammary carcinoma that is IL-6 deficient in vivo (in the mouse 4T1 model); (2) upon further analysis, 4T1 tumors abundantly expressed FcεRI; (3) 4T1 cells and some human breast cancer cell lines abundantly expressed FcεRI in vitro; (4) the function of FcεRI was limited to cytokine production only in some human breast cancer cell lines in vitro; and (5) conversely to cell models, primary human breast cancers probably do not express more FcεRI than primary non-cancerous breast tissue. Importantly, the functionality tests run during this study showed that FcεRI was nonfunctional in BC models in a traditional sense with respect to MCs; to determine function in these cell lines, we would need to examine the signaling pathway of FcεRI in more detail. However, within human breast cancer tissue, FcεRI mRNA expression was downregulated, which means the issue with these cells expressing this receptor could be that they are immortalized, and thus FcεRI expression is an artifact of their natural history that is irrelevant to the real human pathology. This discordance could have arisen due to mutation likely developed by the cells when they were initially immortalized. Interestingly, all the examined BC lines produced FcεRI (perhaps except MCF7), which could indicate another common route to aberrant protein expression in cell lines that might be of future interest. Regardless, these cells should be used cautiously if studying the interaction between BC and MCs within the tumor microenvironment. This caveat is particularly important in the context of 4T1 cells since these are also used as an immunocompetent in vivo BC model in BALB/c mice. These observations also bring forth a call to examine the associations and roles of MCs more intentionally in primary human breast tissue as well as in primary human BC. FcεRI remains a relatively unique surface target for MCs that can be leveraged therapeutically if MCs make any deleterious contributions to the BC microenvironment.

## 5. Conclusions

This study reports that within the cell lines tested, FcεRI was present but partially non-nonfunctional, and when put in the context of human tissue, the overexpression of FcεRI was not translationally relevant. In the future, other BC cell lines could be tested for FcεRI expression and function, as well as for FcεRI protein in primary BC stroma. Looking further down the signal transduction pathway of this receptor to examine expected routes of activation via BTK, Fyn/Lyn, STAT5, etc. might be of interest. However, these directions may not be worth pursuing due to the lack of strong evidence that this receptor is present within real human BC tissue compared to what we observed in the immortalized cell lines.

## Figures and Tables

**Table 1:** FcεRI PCR Primer Sequences (F = forward; R = reverse).

Primer Name	5′-3′ Sequence	Primer Length
FceRIa-F	ACTGTACGGGCAAAGTGTGG	81
FceRIa-R	ACTTCTCACGCGGAGCTTTT	81
FceRIb-F	CCTCCAGTGCACCTGACATT	149
FceRIb-R	ATGTCCGCCATGTCTGCTTT	149
FceRIg-F	GCCGTGATCTTGTTCTTGCTC	78
FceRIg-R	GCCTTTCGGACCTGGATCTT	78

**Table 2:** R2 Genomic tissue samples [16,17,18,19,20,21].

Author Name	Tissue Type	Sample Size
Belitskaya-Levy	Postmenopausal Normal Breast	107
Russo	Nulli-parous Normal Breast	113
Russo	Full-term Pregnancy Normal Breast	109
Gruvberger-Saal	Primary Tumor Breast	3207
Brown	TNBC Tumor Breast	198
Sinn	Tumor Breast Metastatic	1108

## Data Availability

The original contribution presented in the study is included in the article.
